# Manual for the Medical Practitioner

**Published:** 1855-02

**Authors:** 


ART. VI.—*Manual for the Medical Practitioner; including Medical and Pharmaceutical Instructions, and* 541 *Prescriptions.* By G. ARINK, M. D. Rochester: Erastus Darrow and Brother. 1855.

We are at a loss to discover the practical—or theoretical—value of this book. In its typography it does no honor to its publishers, and its proof-reader was not the most careful of men. Its Latin, also, would bear correction. We make the following extracts:

"Dangerous or Deadly Intermittent Fevers.

"*Febres Intermittens Maligna sive Perniciosa.*

"These fevers are called dangerous or deadly intermittent fevers, because every commencement of fever brings the patient in great danger. It is necessary to suppress the fever immediately, and this can be done with Nos. 60, 61 and 62. If the patient has a plethoric constitution, make a *Venae Sectio.* After the fever is suppressed, seek to ascertain the cause of the fever. If it is gastric, bilious, mucous, or worms, then see Gastric, Bilious, and Worm Fevers."

Saying nothing about the latinity of "febres intermittens," we would suggest that it would be as well to "ascertain the cause of the fever" *before* "it is suppressed." The No. 60 referred to is as follows, Nos. 61 and 62 being nearly the same:

"Pulv. cort. chinæ regii, drachm iv.

Opii crudi, gr. ij.

Misce fiant pulv. divid. in ij partes æquales.

"S. One powder to be taken one hour, and the other one two hours before the fever commences."

Which one first?

We are sorry that this book has been forced upon our notice. The author is evidently a painstaking and intelligent man, subjecting himself to misconception by writing in a language not his own. Again, he has evidently mistaken the uses of medical literature in this essay at authorship, and has, we fear, opened the way to much personal mortification. H.

